# Care Pathway and Outcomes in Pediatric Septic Shock: A Narrative Review from Emergency Department Recognition to PICU Management

**DOI:** 10.3390/children13050622

**Published:** 2026-04-30

**Authors:** Efrossini Briassouli, George Briassoulis

**Affiliations:** 1 Second Department of Paediatrics, Aglaia Kyriakou Children’s Hospital, School of Medicine, National and Kapodistrian University of Athens, 10679 Athens, Greece; briasouli@aglaiakyriakou.gr; 2 Postgraduate Program “Emergency and Intensive Care in Children, Adolescents and Young Adults”, School of Medicine, University of Crete, 71003 Heraklion, Greece

**Keywords:** pediatric, septic shock, care pathway, emergency recognition, PICU, prognostic tools

## Abstract

**Highlights:**

**What are the main findings?**

- Pediatric septic shock is best understood as a dynamic hospital trajectory, beginning with early recognition and first-hour treatment and continuing through ward surveillance, PICU escalation, intensive care management, and recovery.
- Outcomes depend not only on PICU therapies, but also on timely antimicrobial treatment, individualized fluid and vasoactive support, repeated reassessment, and prompt escalation across the full continuum of care.

**What are the implications of the main findings?**

- Clinicians should approach pediatric septic shock through a continuum-of-care model that links emergency, ward, and intensive care management rather than treating these phases as separate clinical events.
- Prognosis should be evaluated beyond mortality alone and include organ dysfunction burden, duration of organ support, length of stay, and longer-term functional recovery.

**Abstract:**

Background: Pediatric septic shock remains a major cause of morbidity and mortality and requires timely recognition and management across multiple hospital settings. Although intensive care support is critical, outcomes are also influenced by earlier phases of care, including emergency department recognition, first-hour treatment, inpatient monitoring, and timely escalation to the pediatric intensive care unit (PICU). Objective: We aimed to review pediatric septic shock across the full hospital trajectory, from emergency department recognition to PICU management and outcomes, with emphasis on diagnostic challenges, early treatment, escalation of care, and prognostic assessment. Methods: This narrative review was based on a structured literature search of PubMed/MEDLINE, Scopus, and the Cochrane Library, with emphasis on international guidelines, consensus statements, systematic reviews, and clinically relevant pediatric studies addressing recognition, resuscitation, escalation, intensive care management, and outcomes in pediatric septic shock. Results: Pediatric septic shock is best approached as a dynamic continuum rather than a single event. Early recognition is complicated by age-dependent physiology, nonspecific presentation, and delayed hypotension. Timely antimicrobial therapy, individualized fluid resuscitation, early vasoactive support, and repeated reassessment during the first hours are central to management. Ward surveillance and prompt escalation to PICU are critical, as delayed recognition of deterioration may worsen organ dysfunction and resource use. In the PICU, phenotype-informed hemodynamic support, fluid stewardship, respiratory support, and organ support are essential. Outcomes should be evaluated beyond mortality to include organ dysfunction burden, duration of support, length of stay, and longer-term functional recovery. Conclusions: Pediatric septic shock outcomes are shaped by the entire hospital care pathway rather than PICU treatment alone. A trajectory-based, continuum-of-care approach may improve timely diagnosis, escalation, and short- and longer-term outcomes.

## 1. Introduction

Pediatric septic shock remains a major cause of morbidity, mortality, and healthcare utilization worldwide despite advances in antimicrobial therapy, hemodynamic support, organ support, and critical care delivery [1]. Contemporary epidemiologic data indicate that sepsis continues to impose a substantial burden on infants and children across diverse healthcare settings [2]. Importantly, its impact extends beyond survival alone, as survivors may experience prolonged hospitalization, organ dysfunction, impaired functional recovery, and reduced quality of life [1,2]. These considerations support viewing pediatric septic shock not only as an acute resuscitation emergency but also as a condition with significant downstream consequences.

A major challenge in children is that septic shock often evolves along a dynamic clinical continuum rather than presenting as an immediately obvious syndrome. Because age-dependent physiology may preserve arterial pressure until relatively late in the course of circulatory failure, hypotension is often a late finding [3]. Earlier manifestations may include tachycardia, abnormal perfusion, altered mental status, oliguria, and rising lactate [4] and other laboratory indicators of evolving systemic inflammation [5]. This makes early recognition particularly difficult in the emergency department (ED), where sepsis may initially resemble more common pediatric conditions such as dehydration, viral illness, bronchiolitis, or asthma. Repeated bedside reassessment and careful interpretation of diagnostic support tools are therefore essential [6].

The conceptual framework of pediatric sepsis has also evolved substantially. The 2024 international consensus criteria moved pediatric sepsis away from the older systemic inflammatory response syndrome (SIRS)-based model toward a definition centered on infection-associated organ dysfunction, operationalized through the Phoenix Sepsis Score [7]. Within this framework, septic shock is defined as sepsis accompanied by cardiovascular dysfunction, including severe age-adjusted hypotension, markedly elevated lactate, or vasoactive requirement [7,8]. These updated criteria represent an important advance for epidemiology, research, and severity stratification. However, bedside care still depends on recognizing evolving physiologic instability before formal criteria are fully met.

Current management recommendations emphasize that outcomes in pediatric septic shock are strongly influenced by time-sensitive care [9]. The updated 2026 Surviving Sepsis Campaign international guidelines for children further reinforce this trajectory-based approach by emphasizing hospital performance improvement programs, early blood lactate measurement, prompt antimicrobial therapy, cautious crystalloid-based resuscitation with reassessment after each bolus, and structured hemodynamic evaluation, including cardiac and lung point-of-care ultrasound where expertise is available [10]. These recommendations also acknowledge that, despite important progress, the quality of pediatric evidence remains limited for many clinical questions. In parallel, recent reviews have highlighted increasing interest in individualized management, including hemodynamic phenotyping [11,12], fluid stewardship after initial resuscitation [13], and biomarker- or machine learning-informed risk stratification [14].

Importantly, pediatric septic shock is not solely a PICU disease. A substantial proportion of children who ultimately require intensive care are first recognized and treated outside the PICU, including in the ED, inpatient wards, transport systems, and referring institutions [15,16]. In addition, some children develop sepsis after hospital admission, including in pediatric, surgical, oncology, or other specialty wards, sometimes in the setting of hospital-acquired infection [15,17]. These observations highlight that prognosis may depend not only on intensive care management itself, but also on the timeliness of recognition, initial stabilization, monitoring intensity, and escalation decisions across the hospital system [15,16].

This broader perspective is clinically important because the burden of pediatric septic shock extends beyond mortality alone. Greater organ dysfunction, prolonged vasoactive and ventilatory support, longer PICU and hospital stay, and post-sepsis morbidity are increasingly recognized as meaningful outcome domains [18]. Biomarkers such as lactate may assist with early risk stratification, and emerging diagnostic and prognostic tools may eventually help identify children at highest risk for deterioration earlier in their course [14,19]. Nevertheless, no single marker or tool can replace the integration of bedside recognition, inpatient reassessment, and PICU-based optimization within a coherent care pathway.

Accordingly, this narrative review examines pediatric septic shock as a continuous hospital pathway—from early recognition in the emergency department or inpatient ward to PICU management and outcomes—with emphasis on time-critical decisions, escalation of care, and the factors most likely to influence short- and longer-term outcomes.

## 2. Materials and Methods

### 2.1. Study Design

This article was conducted as a narrative review aimed at providing a clinically focused synthesis of the literature on pediatric septic shock across the hospital care pathway, from early recognition to PICU management and outcomes. The review was structured around the major stages of care, including contemporary definitions and conceptual frameworks, emergency department recognition, early treatment and reassessment, ward monitoring and escalation to PICU, intensive care management, and short- and longer-term outcomes.

### 2.2. Data Sources and Search Strategy

A structured literature search was performed in PubMed/MEDLINE, Scopus, and the Cochrane Library to identify relevant English-language articles. Particular emphasis was placed on studies published between 2020 and March 2026, although earlier landmark publications were also included when they were considered important for historical context, conceptual development, or interpretation of current practice. The search strategy combined terms related to pediatric sepsis and septic shock with terms reflecting the different stages of the hospital trajectory and the main diagnostic and therapeutic domains. Search terms included combinations of “pediatric sepsis,” “pediatric septic shock,” “children,” “emergency department,” “recognition,” “screening,” “ward deterioration,” “escalation of care,” “PICU,” “hemodynamic support,” “outcomes,” “biomarkers,” and “prediction models.” The literature search and initial screening were conducted independently by E.B. and G.B. Titles and abstracts were screened for relevance to the predefined scope of the review, and full-text articles were subsequently assessed when considered potentially eligible or of high clinical relevance. Disagreements regarding study relevance, inclusion, or interpretation were resolved through discussion and consensus between the two authors.

### 2.3. Eligibility Criteria

**Figure 1:**
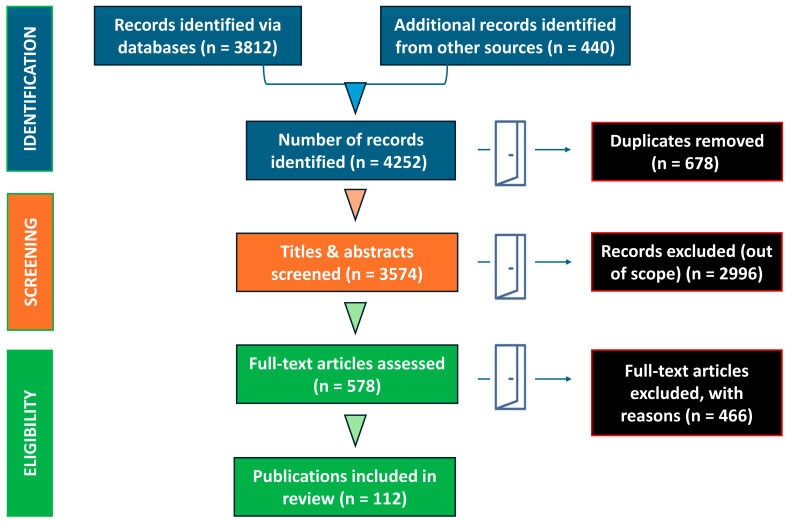
Illustrative PRISMA-like flow diagram of study selection for this narrative review.

Eligible sources included international guidelines, consensus statements, systematic reviews, scoping reviews, narrative reviews, randomized clinical trials, and observational studies, with priority given to pediatric-specific evidence whenever available. Selected adult studies were also considered when they informed broader conceptual, diagnostic, or therapeutic issues in areas where pediatric evidence remained limited. Preference was given to recent publications and to studies with direct clinical relevance to recognition, hemodynamic monitoring, early treatment, escalation of care, PICU management, and outcomes in pediatric septic shock. Reference lists of key publications were also manually screened to identify additional relevant studies not retrieved through the primary database search. Studies were excluded if they were case reports, small case series, conference abstracts, non-peer-reviewed preprints, letters without substantial original data, animal-only studies, or non-English publications. Because this was a narrative rather than a systematic review, study selection was guided primarily by clinical relevance and methodological contribution, rather than by a predefined framework for quantitative synthesis. The study selection process is summarized in an illustrative PRISMA-like flow diagram (Figure 1).

### 2.4. Synthesis and Appraisal

The selected literature was synthesized thematically according to the major stages of care across the hospital trajectory. Particular emphasis was placed on diagnostic timeliness, early management, serial reassessment, escalation to higher-level care, PICU treatment, and outcome determinants. Because the included studies were heterogeneous with respect to populations, definitions, screening tools, monitoring strategies, therapeutic interventions, and reported outcomes, formal quantitative pooling was not appropriate. In appraising the evidence, greater weight was assigned to original studies, higher-level evidence, international guidelines, and findings with clear clinical interpretability and patient-centered relevance. Adult studies were used cautiously and only when pediatric data were sparse or unavailable. Accordingly, the present review should be interpreted as a clinically focused thematic synthesis rather than as a quantitative estimate of intervention effects.

### 2.5. Limitations

This review has several limitations. First, as a narrative rather than systematic review, it remains susceptible to selection bias and publication bias despite the use of multiple databases, structured searching, and manual citation tracking. In addition, the restriction to English-language publications and the exclusion of preprints may have limited the inclusion of potentially relevant data, particularly from low- and middle-income settings and from rapidly emerging areas of the literature. Second, a substantial proportion of the pediatric septic shock literature is based on observational studies, which are vulnerable to confounding by illness severity, treatment timing, clinician preference, and center-level practice variation, thereby limiting causal inference. Third, the available evidence is heterogeneous in terms of definitions, study populations, diagnostic tools, treatment strategies, and reported outcomes, which restricts direct comparison across studies and precludes formal meta-analysis. Accordingly, the present review should be interpreted as a thematic and clinically focused synthesis rather than as a source of definitive effect-size estimates for specific interventions such as fluid volume strategies or vasoactive choices. In addition, for some clinically relevant domains, including fluid stewardship, fluid creep, escalation pathways, and phenotype-informed management, pediatric-specific quantitative thresholds and operational frameworks remain incompletely defined. Finally, in several clinically important areas, including escalation pathways, hemodynamic phenotyping, and long-term outcomes, high-quality pediatric data remain limited, requiring cautious interpretation and, in selected areas, partial reliance on indirect evidence from adult studies.

## 3. Definitions and Conceptual Framework

### 3.1. Definitions of Pediatric Sepsis and Septic Shock

Definitions of pediatric sepsis and septic shock have evolved substantially over the past two decades. The 2005 International Pediatric Sepsis Consensus Conference framework relied largely on systemic inflammatory response syndrome (SIRS) criteria in the presence of suspected or proven infection, but this approach was increasingly criticized for limited specificity and for poor alignment with the contemporary concept of infection-associated organ dysfunction [20].

In 2024, the Society of Critical Care Medicine Pediatric Sepsis Definition Task Force introduced updated international consensus criteria centered on the Phoenix Sepsis Score, thereby shifting pediatric sepsis toward an organ dysfunction-based framework [7]. In this model, sepsis is identified in children with suspected infection and a Phoenix Sepsis Score of at least 2 points, reflecting potentially life-threatening dysfunction in the respiratory, cardiovascular, coagulation, and/or neurologic systems. Septic shock is defined as sepsis accompanied by cardiovascular dysfunction, operationalized by at least 1 cardiovascular point in the Phoenix framework [9].

This conceptual shift is important because it better reflects the pathobiology and clinical severity of pediatric sepsis than SIRS-based definitions and improves predictive validity for adverse outcomes across diverse healthcare settings. The Phoenix criteria were derived and validated in large international pediatric datasets and demonstrated higher positive predictive value, with comparable or improved sensitivity relative to older pediatric consensus criteria [21].

### 3.2. From Static Criteria to Dynamic Clinical Trajectories

Although updated consensus definitions improve research standardization and epidemiologic comparability, bedside recognition of pediatric septic shock remains inherently dynamic. Children often maintain arterial pressure until relatively late in the course of circulatory failure because of robust compensatory vasoconstriction and tachycardia [5]. As a result, significant tissue hypoperfusion may already be present before hypotension develops. Septic shock in children should therefore be understood as a process of evolving physiologic instability rather than a diagnosis that is always captured early by a single threshold-based definition [6].

In practical terms, this means that a child may already be on the septic shock trajectory before all formal diagnostic elements are fulfilled. Repeated assessment of perfusion, capillary refill, mental status, urine output, lactate trends, and respiratory effort remains essential, particularly in emergency and ward settings where deterioration may occur over hours rather than minutes. Definitions support recognition and classification, but they do not replace longitudinal clinical judgment [9].

### 3.3. Why Pediatric Septic Shock Remains Diagnostically Challenging

Pediatric septic shock remains diagnostically challenging because its presentation is heterogeneous and strongly age dependent. Infants and young children may initially present with nonspecific features such as irritability, poor feeding, tachypnea, lethargy, or reduced interaction, which overlap with many common pediatric illnesses. Even when infection is suspected, evolving circulatory dysfunction may not be immediately recognized, especially while blood pressure is still preserved. Scoping reviews of sepsis recognition in pediatric emergency settings have highlighted persistent diagnostic difficulties, including inconsistent screening performance and reliance on signs that may appear relatively late in the disease course [22].

In addition, pediatric septic shock is hemodynamically heterogeneous. Children may present with vasodilatory shock, myocardial dysfunction, mixed shock states, or progressive multiorgan dysfunction, and these phenotypes may evolve over time [23]. This heterogeneity complicates both diagnosis and treatment and partly explains why uniform management strategies often perform inconsistently across populations. Recent reviews of pediatric hemodynamic support therefore emphasize individualized reassessment rather than fixed one-size-fits-all treatment models [24].

### 3.4. Practical Implications for a Hospital-Trajectory Model

Taken together, current evidence supports understanding pediatric septic shock as a hospital trajectory rather than a single event. The Phoenix criteria provide an important research and clinical framework for identifying infection-associated organ dysfunction, but children are often recognized, treated, monitored, and escalated across multiple care environments before or after formal criteria are met. Although the Phoenix framework improves contemporary standardization of pediatric sepsis and septic shock, some of its components may not be immediately available in non-ICU or resource-constrained settings. Accordingly, bedside recognition must still rely on serial clinical assessment and early identification of evolving organ dysfunction even before complete score ascertainment is possible. The dynamic nature of pediatric shock, therefore, reinforces the need to integrate emergency department recognition, inpatient monitoring, PICU escalation, and intensive care reassessment within a single continuum of care [7]. The pediatric Surviving Sepsis Campaign is aligned with this trajectory-based perspective, emphasizing serial reassessment, time-sensitive interventions, and context-specific adaptation across the pathway of care [9]. To summarize the contemporary conceptual framework and its bedside implications, the main definitions relevant to pediatric septic shock are presented in Table 1.

## 4. Recognition in the Emergency Department

Early recognition of pediatric septic shock in the emergency department (ED) remains both a critical and a challenging component of the overall care pathway. Children often present before overt cardiovascular collapse, while early manifestations are frequently nonspecific, such as fever, lethargy, irritability, poor feeding, tachypnea, vomiting, or reduced interaction. Unlike in adults, arterial hypotension is typically a late finding in children, because compensatory vasoconstriction and tachycardia may initially preserve blood pressure despite significant circulatory compromise.

Recognition in the ED, therefore, relies on a combination of clinical suspicion, repeated bedside assessment, and timely physiologic reassessment [25]. Early indicators of evolving shock may include altered mental status, prolonged or abnormally brisk capillary refill, cool or mottled extremities, weak peripheral pulses, tachycardia disproportionate to fever, oliguria, and increased work of breathing [26]. Within this framework, pediatric septic shock should be understood not as a single diagnostic event, but as a dynamic process of progressive physiologic instability.

The substantial overlap between early septic shock and common pediatric ED presentations, including dehydration, bronchiolitis, asthma exacerbation, gastroenteritis, diabetic ketoacidosis, and viral illnesses, further increases diagnostic uncertainty. A comparative study of pediatric sepsis identification in emergency departments demonstrated considerable heterogeneity in screening strategies and persistent difficulty differentiating sepsis from other febrile conditions, particularly in younger children [27]. Although screening tools and early warning systems may improve triage awareness and promote standardized evaluation, their diagnostic performance remains imperfect. They should therefore be used to support, rather than replace, clinical judgment [28].

Laboratory evaluation represents an important adjunct to clinical assessment. Initial investigations commonly include blood gas analysis, lactate, complete blood count, inflammatory markers, renal and liver function tests, coagulation studies, and microbiologic cultures when feasible. Among these variables, lactate has attracted particular interest as a marker of illness severity, tissue hypoperfusion, and outcome risk [26,29]. Nevertheless, elevated lactate is neither specific for septic shock nor consistently present in all cases. For this reason, serial lactate trends are generally more informative than isolated measurements, and current pediatric sepsis guidance recommends interpreting lactate within a broader framework of repeated clinical reassessment rather than as a stand-alone diagnostic marker [9].

Recognition is also influenced by the surrounding system of care. Standardized sepsis screening processes, electronic alerts, rapid escalation pathways, and bundle-based triage responses may reduce delays to treatment, although their effectiveness varies according to local organization, staffing, and available resources. Importantly, recognition should not be viewed as complete after the initial triage encounter [30]. A child who does not fully meet sepsis criteria at presentation may deteriorate over the following hours, making serial reassessment an essential element of safe emergency care. This is particularly relevant in children who remain under ED observation or who are admitted to the pediatric ward after an initial period of apparent stabilization [31].

From a practical standpoint, ED recognition of pediatric septic shock may be structured around a series of key bedside questions: Is infection likely? Are there signs of impaired perfusion or evolving organ dysfunction? Is the child improving, remaining unchanged, or deteriorating on serial reassessment? And does the current level of care remain appropriate? This trajectory-based approach is clinically more useful than reliance on any single sign, laboratory value, or screening threshold [32]. It is also consistent with the contemporary shift toward infection-associated organ dysfunction and time-sensitive escalation of care in pediatric sepsis.

Overall, the literature supports the view that recognition in the ED is not simply a matter of assigning the label of sepsis, but of identifying the child who is entering a septic shock pathway and therefore requires immediate treatment, close monitoring, and possible escalation to higher-level care. Timely recognition enables earlier antimicrobial therapy, more appropriate fluid and vasoactive support, and earlier PICU involvement when needed, thereby influencing outcomes well before intensive care begins [33].

Importantly, early recognition of pediatric sepsis should not be framed exclusively as an emergency department challenge. Although many children present from the community and are first assessed in the ED, others develop sepsis later during hospitalization, including on pediatric, surgical, oncology, or other specialty wards, either because an initially localized infection progresses or because a hospital-acquired infection emerges. This distinction is clinically relevant, since delayed recognition may be even more likely in already hospitalized children with postoperative status, malignancy, immunosuppression, invasive devices, or complex underlying disease. Therefore, the principles of early identification, serial reassessment, and timely escalation of care apply across the entire hospital pathway and should not be restricted to the point of entry into the healthcare system [6,7].

## 5. Early Management During the First Hours

**Figure 2:**
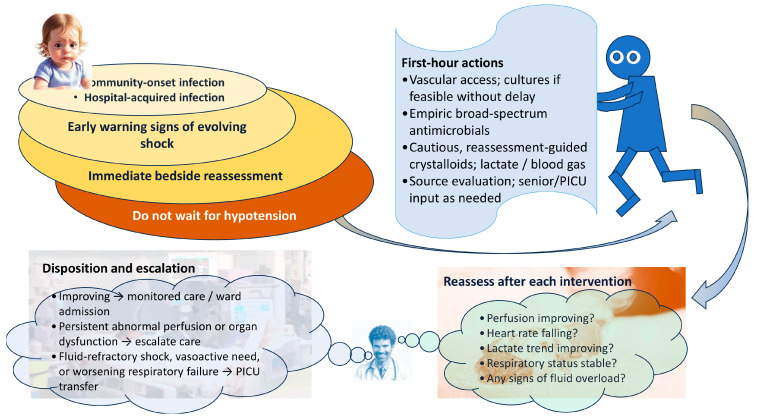
Recognition and first-hour management pathway in pediatric septic shock.

The first hours of treatment should likewise be considered within a broader hospital context rather than as a phase limited to emergency care. In some children, sepsis is recognized immediately on presentation to the ED; in others, it evolves during inpatient hospitalization and becomes apparent only after clinical deterioration on the ward. Accordingly, timely antimicrobial therapy, hemodynamic stabilization, repeated reassessment, and decisions regarding PICU referral are relevant both for community-onset and hospital-onset sepsis. A practical recognition and first-hour management pathway for pediatric septic shock is shown in Figure 2.

### 5.1. Early Antimicrobial Therapy

Prompt administration of empiric antimicrobial therapy remains a cornerstone of pediatric sepsis management. Once sepsis or septic shock is suspected, treatment should not be delayed while awaiting full diagnostic confirmation, particularly in children with signs of impaired perfusion or evolving organ dysfunction. Current pediatric guidance supports the initiation of broad-spectrum antimicrobials as early as possible after recognition, while also emphasizing that therapy should be guided by the likely source of infection, prior antimicrobial exposure, comorbidities, immunologic status, and local microbiologic epidemiology [34]. The 2026 pediatric Surviving Sepsis Campaign guidelines recommend antimicrobial administration as soon as possible, ideally within 1 h of recognition in children with suspected septic shock. In children with probable sepsis without shock, they suggest a rapid time-limited evaluation followed by antimicrobial initiation as soon as possible, ideally within 3 h, if concern for sepsis is substantiated [10]. In practice, this means that the initial choice of antimicrobials should balance urgency with adequacy of coverage, especially in children at risk for resistant organisms, healthcare-associated infection, central line-related infection, or invasive fungal disease.

At the same time, timely antimicrobial therapy should not be equated with indiscriminate antimicrobial use. Blood cultures and other relevant microbiologic samples should be obtained before antimicrobial administration whenever feasible, provided this does not substantially delay treatment [10]. Once culture results, molecular diagnostics, or further clinical information become available, antimicrobial therapy should be narrowed, discontinued, or redirected accordingly. Thus, the early phase requires both speed and stewardship: rapid initiation of appropriate empiric therapy followed by structured reassessment and de-escalation where possible.

### 5.2. Fluid Resuscitation: How Much, How Fast, and for Whom

Initial fluid therapy remains central to early resuscitation, but it should be individualized and repeatedly reassessed. Contemporary pediatric recommendations increasingly support a cautious, physiology-guided approach rather than indiscriminate repeated bolus administration. In children with septic shock, crystalloids are recommended for initial resuscitation, with balanced or buffered crystalloids preferred over 0.9% saline when available, and aliquots of approximately 10 mL/kg may be administered with frequent reassessment of perfusion, respiratory status, hepatomegaly, and signs of fluid accumulation [10,35]. Where intensive care resources are available, cumulative volumes of 40–60 mL/kg in the first hour may still be appropriate in selected patients, provided that close monitoring and reassessment follow each bolus, and that further fluid administration is stopped promptly if shock resolves or fluid overload emerges [10]. This individualized approach is particularly important because excess fluid administration may worsen respiratory mechanics, tissue edema, and organ dysfunction. The growing recognition of fluid creep and fluid overload as clinically relevant contributors to worse outcomes has shifted emphasis from simply giving fluids early to giving the right amount of fluid to the right child, at the right time, with structured reassessment [13,36]. However, validated pediatric septic shock-specific quantitative thresholds for when fluid creep itself becomes clinically harmful remain insufficiently defined. At present, its significance should be interpreted within the broader context of cumulative fluid balance, capillary leak, respiratory status, organ dysfunction, and repeated physiologic reassessment. Accordingly, early fluid management should be seen as a dynamic component of hemodynamic support rather than a fixed-volume protocol.

### 5.3. Early Vasoactive Support

When shock persists despite initial fluid resuscitation, vasoactive support should not be delayed. Increasingly, pediatric practice has moved away from repeated fluid boluses in a child who remains poorly perfused and toward earlier initiation of vasoactive therapy, including peripheral administration when central access is not yet available and institutional expertise permits [9]. This shift reflects the recognition that fluid-refractory shock may develop early and that continued fluid administration in a non-responsive child may increase harm rather than improve perfusion [37].

The decision to begin vasoactive support should be based on the overall hemodynamic picture. Persistent tachycardia, prolonged capillary refill, weak pulses, altered mental status, rising lactate, oliguria, or increasing respiratory distress may all indicate the need for escalation even before overt blood pressure decline occurs. Early vasoactive therapy therefore represents not only a treatment choice, but also a marker of timely recognition that the child’s physiology is failing to respond adequately to fluids alone [38].

### 5.4. Source Control and Antimicrobial Tailoring

Initial stabilization should proceed in parallel with efforts to identify the source of infection and determine whether source control is required [39]. In pediatric sepsis, this may include drainage of abscesses or infected collections, removal or replacement of infected vascular devices, evaluation of abdominal or postoperative sources, and reassessment of potential healthcare-associated infections. Failure to address the source of infection may limit the effectiveness of otherwise appropriate antimicrobial therapy and contribute to ongoing organ dysfunction.

Equally important is the transition from empiric treatment to targeted treatment. As microbiologic data, imaging findings, and clinical evolution become available, therapy should be reassessed and adjusted accordingly [9]. This includes narrowing unnecessarily broad coverage, escalating when resistant organisms are suspected or documented, and reconsidering the diagnosis when the clinical course is atypical. Thus, early management should combine prompt empiric action with disciplined reassessment.

### 5.5. Reassessment During the First 1–6 h

The first hours of treatment should be viewed as a continuous cycle of intervention and reassessment rather than as a single resuscitation episode [40]. This iterative model is strongly aligned with the 2026 pediatric Surviving Sepsis Campaign recommendations, which emphasize repeated clinical evaluation during resuscitation rather than protocolized fluid administration without physiologic reappraisal [10]. Response to therapy should be evaluated using repeated bedside assessment of mental status, heart rate, pulse quality, capillary refill, blood pressure, urine output, respiratory effort, and oxygen requirement, together with selected laboratory trends where available. This repeated reassessment is particularly important in children because early improvement may be incomplete, transient, or misleading.

Among laboratory parameters, lactate may contribute to risk stratification and follow-up, but its interpretation should remain cautious. Elevated lactate is not specific to septic shock and may reflect multiple physiologic mechanisms beyond tissue hypoperfusion. For this reason, the recent literature supports the use of serial lactate trends within the broader context of clinical reassessment rather than reliance on an isolated value [19]. The same principle applies more generally: no single physiologic or laboratory variable should override the overall trajectory of the child.

### 5.6. The Importance of the First Hours for the Later Hospital Trajectory

A practical way to view early treatment is not simply as “ED management,” but as the phase that determines the subsequent hospital pathway. Children who respond promptly may stabilize with ongoing inpatient monitoring, whereas those with persistent perfusion abnormalities, rising lactate, vasoactive requirement, worsening respiratory distress, or evolving organ dysfunction may require urgent PICU escalation [41]. Thus, the quality of care delivered in the first 1–6 h is closely linked to subsequent need for ventilation, organ support, length of stay, and overall outcome [42]. These observations further support the need to conceptualize early management as a hospital-wide, time-sensitive process rather than as an ED-only intervention [43]. The main components of early management across the emergency department, inpatient ward, and PICU are summarized in Table 2.

## 6. Pediatric Ward Monitoring and Escalation to PICU

Not all children with sepsis enter the hospital pathway through the emergency department. Some develop sepsis or septic shock while already hospitalized on pediatric, surgical, oncology, or other specialty wards, either because a localized infection progresses despite treatment or because a hospital-acquired infection emerges during admission [44,45]. This distinction is clinically important, as delayed recognition may be more likely in children with postoperative status, malignancy, immunosuppression, indwelling devices, or complex chronic disease, in whom early signs of deterioration may be subtle or initially attributed to the underlying condition [46]. In this setting, pediatric sepsis surveillance should be understood as a hospital-wide responsibility rather than a process confined to triage or emergency care [47].

### 6.1. Ward Monitoring After Initial Stabilization

After the first phase of treatment, some children show only partial or transient improvement and remain at risk for subsequent deterioration. For this reason, ward monitoring should not be regarded as a passive observation period, but as an active phase of reassessment aimed at identifying persistent or evolving organ dysfunction [48,49]. Repeated bedside evaluation should focus on mental status, heart rate, pulse quality, capillary refill, blood pressure, urine output, respiratory effort, oxygen requirement, and, where available, relevant laboratory trends such as lactate. The objective is not merely to document vital signs, but to determine whether perfusion and organ function are truly improving [50].

A central challenge in ward-based care is that deterioration often precedes overt cardiovascular collapse. Persistent tachycardia, abnormal capillary refill, worsening peripheral perfusion, increasing oxygen demand, altered consciousness, oliguria, or rising lactate should prompt immediate clinical reassessment and reconsideration of the current level of care. Importantly, the absence of hypotension should not delay escalation when the overall trajectory suggests unresolved shock or progressive organ dysfunction [51].

### 6.2. Detection of Deterioration and Escalation Pathways

Structured systems for identifying deterioration may support earlier recognition, but they should complement rather than replace clinical judgment. Pediatric early warning systems (PEWS), rapid response pathways, and standardized escalation algorithms can improve situational awareness and may facilitate more timely PICU consultation or transfer [28]. However, their performance varies across institutions, populations, and implementation strategies, and recent literature continues to emphasize the need for local validation and appropriate staff training [52]. Accordingly, these tools are most useful when embedded within a broader culture of repeated bedside assessment, timely senior review, and low-threshold escalation for children whose condition is not clearly improving [53].

From a practical perspective, monitoring on the ward should repeatedly address three questions: Is the child responding to the initial therapeutic strategy? Is new or progressive organ dysfunction emerging? And does the child now require PICU-level support? Framing surveillance in this way helps translate bedside findings into timely action and reduces the risk that deterioration will be recognized only after advanced decompensation has occurred [54].

### 6.3. Indications for PICU Consultation and Transfer

Data from hospitalized children requiring unplanned PICU transfer indicate that deterioration on the ward is often preceded by a recognizable phase of clinical decline and may be associated with substantial morbidity and mortality, particularly in children with underlying comorbidity, including oncology patients [46]. The transition from ward-based care to PICU is a particularly vulnerable phase in the management pathway. Available pediatric deterioration and transfer literature suggests that delayed escalation is associated with greater physiologic derangement, increased resource use, and worse outcomes, even when the initial signs of decline appear modest [41,46,55]. This is especially relevant in children with sepsis, in whom progression may be rapid, non-linear, and initially difficult to distinguish from the course of the underlying disease [41].

PICU consultation or transfer should therefore be considered when there is persistent or worsening perfusion abnormality, need for vasoactive support, fluid-refractory shock, escalating respiratory failure, rising burden of organ dysfunction, or uncertainty that ward-level monitoring can provide sufficiently close surveillance [42]. Decisions should be based on the child’s overall trajectory. In practice, this means that a child who remains tachycardic, poorly perfused, increasingly hypoxemic, oliguric, or neurologically altered despite initial treatment should not remain on the ward simply because blood pressure is temporarily maintained [9].

Practical bedside triggers that should prompt PICU consultation or transfer include persistent abnormal perfusion despite initial treatment, rising lactate, increasing vasoactive requirement or anticipated need for vasoactive support, escalating respiratory support, worsening mental status, oliguria, recurrent need for fluid boluses, or concern that the frequency of reassessment required cannot be safely delivered at ward level.

### 6.4. Clinical Meaning of Timely Escalation

**Figure 3:**
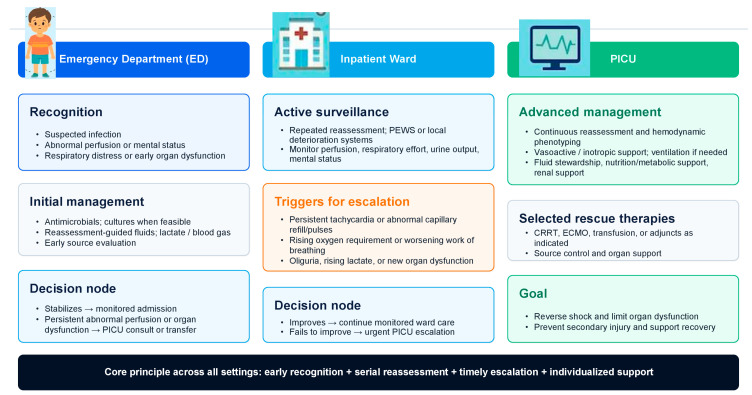
Escalation-of-care pathway from the emergency department and inpatient ward to the PICU.

Timely escalation to PICU should not be interpreted as failure of ward management, but as an appropriate response to an evolving disease process. The quality of sepsis care depends not only on the correctness of the initial interventions, but also on how efficiently the system detects non-response and adapts the level of support [50]. In this regard, the ward-to-PICU transition represents a continuation of early sepsis care rather than a separate phase. Figure 3 summarizes the escalation-of-care pathway from ED or inpatient ward recognition to PICU transfer when closer monitoring or organ support is required. In this sense, timely escalation should be viewed as an integral component of high-quality sepsis care rather than as a delayed response to established decompensation.

## 7. PICU Management

Once pediatric septic shock progresses to the PICU, management shifts from initial stabilization to continuous reassessment, phenotype-informed hemodynamic support, organ support, and prevention of secondary injury [49]. Current pediatric guidance emphasizes that the goal is not simply normalization of blood pressure, but restoration of adequate tissue perfusion and limitation of ongoing organ dysfunction [56]. The Surviving Sepsis Campaign pediatric guidelines recommend using advanced hemodynamic variables, when available, in addition to bedside clinical variables, and using lactate trends alongside clinical assessment to guide ongoing resuscitation [9].

### 7.1. Hemodynamic Phenotypes and Cardiovascular Dysfunction

Pediatric septic shock is hemodynamically heterogeneous, with children presenting with vasodilatory shock, myocardial dysfunction, mixed shock states, or progressive multiorgan dysfunction. Because these phenotypes may evolve over time, repeated clinical and hemodynamic reassessment is essential, and reliance on bedside signs alone may be insufficient to guide individualized therapy [57]. Recent reviews therefore caution against relying on bedside clinical signs alone to categorize shock as “warm” or “cold” and favor a more integrated hemodynamic assessment. This is consistent with guideline recommendations that bedside signs alone should not be used in isolation for shock categorization [24].

In settings without advanced monitoring, bedside differentiation of vasodilatory, myocardial, or mixed shock phenotypes remains provisional. Serial assessment of pulse quality, capillary refill, extremity temperature, urine output, blood pressure, hepatomegaly, mental status, and lactate trends may raise suspicion of different hemodynamic patterns, but echocardiography and point-of-care ultrasound provide a more reliable basis for phenotype-informed management when available.

### 7.2. Advanced Monitoring and Repeated Reassessment

PICU management relies on serial reassessment of perfusion, organ dysfunction, and treatment response. Beyond bedside variables such as heart rate, capillary refill, urine output, blood pressure, and mental status, advanced hemodynamic monitoring may include cardiac output/cardiac index, systemic vascular resistance, central venous oxygen saturation, and bedside echocardiography where available [58]. Emerging pediatric evidence also supports the increasing role of point-of-care echocardiography in characterizing hemodynamic phenotype and guiding individualized fluid and vasoactive management [59]. Consistent with this approach, the 2026 pediatric Surviving Sepsis Campaign guidelines suggest using cardiac and lung point-of-care ultrasound to help guide resuscitation and hemodynamic evaluation, provided appropriate expertise and local resources are available [10].

### 7.3. Vasoactive and Inotropic Therapy

When fluid-refractory shock persists, vasoactive and inotropic support becomes central to PICU management. Current pediatric practice increasingly favors earlier vasoactive escalation over repeated prolonged fluid boluses, especially when perfusion remains abnormal or fluid overload risk is rising [60]. Vasoactive therapy should be individualized according to the dominant hemodynamic disturbance, recognizing that myocardial dysfunction and vasoplegia may coexist and change over time [61,62]. Accordingly, pediatric hemodynamic reviews emphasize titration to therapeutic endpoints rather than fixed drug algorithms alone [24].

### 7.4. Respiratory Support and Mechanical Ventilation

Respiratory failure frequently accompanies pediatric septic shock, either because of primary pulmonary infection, sepsis-associated lung injury, increased work of breathing, or hemodynamic instability [63]. In the PICU, respiratory support may range from supplemental oxygen and noninvasive ventilation to invasive mechanical ventilation. Mechanical ventilation should be regarded not only as respiratory support but also as a hemodynamic intervention, because it may reduce oxygen consumption and work of breathing while also affecting preload, afterload, and cardiac output [64]. Management should therefore aim for adequate gas exchange while minimizing ventilator-induced lung injury and avoiding further hemodynamic compromise [65].

### 7.5. Fluid Stewardship After Initial Resuscitation

After the initial resuscitation phase, the clinical problem often shifts from inadequate intravascular volume to harmful cumulative fluid balance. Emerging pediatric critical care literature increasingly supports a fluid stewardship approach that includes reassessment of maintenance fluids, drug diluents, nutrition-related fluids, diuretic strategies, and timely consideration of de-resuscitation [13]. This is also supported by the 2026 pediatric guidelines, which advise avoiding excessive cumulative fluid administration and considering active fluid removal after hemodynamic stabilization when fluid overload is contributing to ongoing organ dysfunction [10]. Recent pediatric review and meta-analysis show that fluid overload is associated with worse outcomes across critical illness populations and may be particularly relevant in septic shock when combined with respiratory failure, AKI, CRRT, or ECMO [66].

### 7.6. Organ Support and Rescue Therapies

Severe pediatric septic shock may require escalation beyond vasoactive therapy and ventilation to renal replacement therapy, extracorporeal membrane oxygenation (ECMO), transfusion support, and management of coagulation or metabolic derangements [67]. The pediatric Surviving Sepsis Campaign includes recommendations on blood transfusion thresholds, plasma exchange in selected TAMOF-related contexts, and ECMO/renal support considerations, while also acknowledging that evidence quality is low for many advanced interventions [9]. Organ support should therefore be individualized, guided by evolving organ dysfunction burden and local expertise.

### 7.7. Toward Phenotype-Informed and Individualized Care

A recurring message in recent pediatric septic shock literature is that PICU management should be individualized rather than protocolized in a rigid manner [68,69]. The combination of serial clinical examination, advanced hemodynamic assessment, bedside echocardiography, and organ dysfunction trends may help identify which child requires more fluid removal, more vasoactive support, more inotropy, ventilatory adjustment, or rescue organ support [34]. This approach aligns with the broader movement toward precision hemodynamics and repeated therapeutic adaptation in pediatric septic shock [24]. The principal domains of PICU management, together with their practical targets and expected effects, are summarized in Table 3.

## 8. Outcomes Across the Hospital Trajectory

Outcomes in pediatric septic shock should be assessed across the entire hospital pathway rather than only at the point of PICU admission or discharge [1]. Mortality remains a central endpoint, but it captures only part of the disease burden. Increasingly, pediatric sepsis literature emphasizes that outcomes also include organ dysfunction burden, duration of vasoactive and respiratory support, PICU and hospital length of stay, resource use, and longer-term functional recovery [70,71]. This broader perspective is particularly relevant because the effects of delayed recognition, suboptimal early treatment, or late escalation may persist well beyond initial stabilization [21].

### 8.1. Mortality and Short-Term Clinical Outcomes

Mortality in pediatric septic shock varies substantially across settings, reflecting differences in case mix, definitions, resources, and timing of recognition. More severe cardiovascular dysfunction, need for invasive ventilation, acute kidney injury, and greater organ dysfunction burden are consistently associated with worse short-term outcomes [1]. Recent observational studies continue to show that septic shock on PICU admission is associated with substantially higher risk of death than many other PICU admission diagnoses, reinforcing the prognostic importance of early hemodynamic failure [72].

At the same time, mortality alone does not adequately distinguish between children who recover promptly and those who survive after prolonged multiorgan dysfunction and intensive resource use. For this reason, short-term outcome assessment should be interpreted alongside the severity and reversibility of organ failure, the response to early therapy, and the need for escalation across the hospital course [73,74].

### 8.2. Organ Dysfunction Burden and Resource Utilization

For many children, the clinical burden of septic shock is expressed not only through survival but through the intensity and duration of organ support. Prolonged vasoactive requirements, respiratory failure requiring invasive mechanical ventilation, renal dysfunction, coagulopathy, and secondary complications contribute to longer PICU stay and greater healthcare use [74]. Reviews on pediatric sepsis quality improvement and outcomes emphasize that these short-term endpoints are highly relevant because they reflect both illness severity and the effectiveness of time-sensitive care delivered earlier in the trajectory [19].

This is one reason why outcome interpretation should remain linked to the full care pathway. A child whose shock is recognized late or whose deterioration is not promptly escalated may survive, yet still experience greater organ dysfunction burden, longer exposure to invasive support, and increased resource utilization. In this sense, morbidity-related endpoints often reflect system performance more sensitively than mortality alone [71,75].

### 8.3. Functional Outcomes and Post-Sepsis Morbidity

Longer-term morbidity among pediatric sepsis survivors is receiving increasing attention. A 2024 narrative review on long-term outcomes after pediatric sepsis highlighted evidence for impairments across physical, cognitive, emotional, and social domains, framed within the concept of post-intensive care syndrome in pediatrics (PICS-p) [75]. More recent data also support that some children surviving sepsis experience subsequent cognitive or developmental difficulties and reduced quality of life, even when hospital survival is achieved [70,71].

These observations are clinically important because they shift the focus from survival alone to recovery quality. Functional outcome, neurodevelopmental trajectory, and post-discharge well-being should increasingly be considered part of the outcome spectrum of pediatric septic shock rather than separate downstream issues [76].

### 8.4. Which Stages of the Hospital Trajectory Most Influence Outcome?

A key implication of the available literature is that outcome is cumulative rather than location-specific. The child’s trajectory may begin with recognition and first-hour treatment in the emergency department, but it may also start after hospital admission, when sepsis evolves on a pediatric, surgical, oncology, or other inpatient ward, including in the context of hospital-acquired infection [45]. It then continues through ward surveillance and escalation decisions and culminates in PICU hemodynamic and organ support. Delays in recognition, escalation, or delivery of definitive intensive care at any point along this pathway may contribute to worsening organ dysfunction and greater resource use, even if ICU-level care is later provided [77]. This is consistent with contemporary pediatric sepsis literature, which increasingly frames prognosis as the product of timely diagnosis, repeated reassessment, effective transitions of care, and early management across the acute care continuum [78].

### 8.5. Practical Interpretation

Taken together, these data support a trajectory-based view of pediatric septic shock outcomes [79]. Survival alone is no longer sufficient to define success. Instead, outcomes should be considered across multiple domains: rapid shock reversal, limitation of organ dysfunction, reduced need for prolonged invasive support, shortened PICU and hospital stay, and preservation of longer-term function and quality of life [80]. This broader framework is particularly useful for a review focused on the full hospital pathway, because it links emergency recognition, inpatient reassessment, PICU care, and survivorship into a single clinically meaningful continuum [76]. The main outcome domains across the hospital trajectory of pediatric septic shock are summarized in Table 4.

## 9. Diagnostic and Prognostic Tools Along the Pathway

Because outcomes in pediatric septic shock are shaped by the entire hospital trajectory, there is growing interest in tools that can improve early recognition, risk stratification, and individualized decision-making across settings [81]. These tools include biomarkers, organ dysfunction scores, bedside hemodynamic assessment, and emerging digital prediction technologies [82,83]. Their principal role is not to replace clinical judgment, but to support earlier identification of children at highest risk for deterioration, persistent organ dysfunction, or death [84].

### 9.1. Prognostic Markers and Evolving Outcome Assessment

Beyond lactate, several inflammatory biomarkers have shown prognostic potential. In adults, soluble urokinase plasminogen activator receptor (suPAR), interleukin-6 (IL-6), and procalcitonin (PCT) have demonstrated value for short-term mortality prediction in sepsis and septic shock [85], while in children, elevated IL-6 and PCT have been associated with increased risk of sepsis among febrile patients [86]. Other markers, such as soluble triggering receptor expressed on myeloid cells-1 (sTREM-1) and presepsin, may provide better diagnostic and prognostic discrimination than conventional inflammatory markers, including C-reactive protein (CRP) and PCT, although broader validation is still needed [87]. Emerging evidence also suggests that sepsis prognosis cannot be fully captured by inflammatory markers alone. Disturbances in oxidative stress, apoptotic and anti-apoptotic pathways, heat shock protein expression, metabolic regulation, and endocrine stress responses have all been linked to disease severity and adverse outcomes [88,89,90,91,92,93]. However, most of these markers remain investigational, and their clinical applicability is limited by small study populations, biologic heterogeneity, and lack of assay standardization [94]. Taken together, current evidence suggests that future prognostic assessment in sepsis will likely depend less on single biomarkers and more on integrated multimarker and physiologic approaches that combine inflammatory, metabolic, immune, and organ dysfunction signals [95,96,97]. Recent reviews therefore support interpreting biomarkers as adjuncts within a broader clinical and physiologic framework rather than as stand-alone decision tools [98].

### 9.2. Clinical Scores and Organ Dysfunction Tools

Severity and organ dysfunction scores remain central to prognostic assessment. The most important recent advance is the Phoenix Sepsis Score, developed and validated in 2024 as part of the new international consensus criteria for pediatric sepsis and septic shock. In children with suspected infection, a Phoenix score of at least 2 points identifies sepsis, while septic shock is defined by sepsis plus at least 1 cardiovascular point [72]. The Phoenix framework was derived from very large international datasets and demonstrated better positive predictive value, with similar or improved sensitivity compared with earlier pediatric sepsis criteria [99]. At the same time, newer external evaluations suggest that Phoenix criteria may perform better for predicting early severe deterioration or death than for broader in-hospital mortality alone, underscoring that even improved scores should still be interpreted within clinical context [100]. Thus, organ dysfunction scores are most useful when they structure assessment, communicate severity, and help track trajectory over time, rather than when they are used as substitutes for bedside judgment.

### 9.3. Hemodynamic and Imaging Tools

In the PICU and, increasingly, in emergency and acute care settings, bedside echocardiography and focused ultrasound may refine hemodynamic assessment by identifying ventricular dysfunction, preload limitation, and mixed shock phenotypes [101]. These tools can support more individualized decisions regarding fluid administration, vasoactive therapy, and escalation of care [102]. Although the evidence remains heterogeneous, recent pediatric studies and reviews support the growing role of imaging-assisted phenotyping as part of precision hemodynamic management [103].

### 9.4. Prediction Models and Digital Technologies

Prediction models and digital tools may ultimately improve early warning, triage, and deterioration detection in sepsis; however, at present they should be viewed as adjunctive rather than stand-alone decision-making systems [84]. Although several models have reported promising AUROC values, these findings should be interpreted cautiously, as many studies were retrospective, single-center, and not externally validated, and few were tested within real-time clinical workflows [84]. Good statistical discrimination in derivation datasets does not by itself ensure transportability, calibration, or meaningful bedside impact [104]. Current evidence therefore highlights the need for multicenter validation, better calibration, transparent reporting, and stronger integration into real-world clinical workflows before routine implementation can be justified [84,105].

### 9.5. Practical Interpretation Across the Hospital Pathway

Across the full hospital trajectory, diagnostic and prognostic tools are most useful when matched to the stage of care. In the emergency department, biomarkers and clinical screening tools may support early suspicion and triage. On the ward, repeated organ dysfunction assessment and deterioration monitoring may help identify children needing escalation. In the PICU, serial hemodynamic, laboratory, and organ dysfunction tools can support phenotype-informed treatment and monitoring of response. The key principle is that no single tool is sufficient; meaningful prognostication arises from integrating clinical examination, trajectory, and structured supportive measures.

## 10. Gaps in Knowledge and Future Directions

Despite major advances in definitions, supportive care, and systems-based management, important gaps remain in the evidence base for pediatric septic shock. One of the central limitations is the continued scarcity of pediatric-specific interventional data, particularly for key questions related to fluid resuscitation, vasoactive choice, escalation thresholds, and advanced hemodynamic support. Much of current practice still depends on observational studies, extrapolation from adult data, or expert consensus, which contributes to persistent variation across institutions and healthcare systems [9,79].

A second major gap concerns the early phases of the hospital trajectory, especially recognition in the emergency department and deterioration on the pediatric ward. Although screening systems, early warning scores, and sepsis bundles are increasingly used, their real-world performance remains variable, and many tools have limited specificity or insufficient external validation in diverse pediatric populations [6,28,52]. Future work should therefore move beyond simple screening toward tools that can better distinguish transient physiologic disturbance from evolving septic shock requiring urgent escalation [84,104]. The 2026 pediatric Surviving Sepsis Campaign guidelines also highlight an important systems-level priority: the implementation of hospital performance improvement programs and standardized operating procedures, underscoring that pediatric sepsis outcomes depend not only on bedside therapies but also on institutional preparedness and coordinated pathways of care [10].

The transition between levels of care also remains insufficiently studied. Delayed PICU transfer, inconsistent escalation criteria, and differences in ward monitoring capacity may all influence outcomes, yet these aspects are often underrepresented in pediatric septic shock research [46,55]. A hospital-trajectory model highlights the need for studies that examine not only what treatments are given, but also when, where, and under what monitoring conditions they are delivered [78,79]. This is particularly important because deterioration may occur gradually and may be modifiable through earlier recognition and faster system response [46,52].

Another important frontier is precision medicine and phenotype-informed management. Pediatric septic shock is biologically and hemodynamically heterogeneous, and future progress will likely depend on better identification of clinically meaningful subgroups [11,81]. Biomarker panels, serial organ dysfunction scoring, bedside echocardiography, and artificial intelligence-assisted prediction models may eventually support more individualized treatment strategies, but most current tools still require stronger multicenter validation and clearer demonstration of clinical utility before widespread implementation [98,101,102,103,104,105].

Longer-term outcomes also deserve greater emphasis. Mortality alone is no longer sufficient to describe the burden of pediatric septic shock. Increasing evidence points to important post-sepsis morbidity, including neurocognitive, physical, emotional, and functional sequelae, yet long-term follow-up remains inconsistently incorporated into pediatric studies [18,76,80]. Future research should therefore include survivorship, quality of life, and family-centered outcomes as core endpoints rather than optional secondary considerations [75,76,80].

Finally, progress in pediatric septic shock will require not only better therapies, but also better integration of care across the hospital pathway. Future studies should more explicitly link emergency recognition, first-hour treatment, ward surveillance, escalation decisions, PICU management, and post-discharge recovery. Such an approach may provide a more realistic and clinically useful understanding of why outcomes differ and where interventions are most likely to improve them [78,79]. In this sense, the next step in pediatric septic shock research may not be a single new biomarker or treatment, but a better ability to connect diagnosis, treatment, escalation, and outcomes within one coordinated continuum of care [78,105].

## 11. Conclusions

Pediatric septic shock is best understood as a dynamic hospital trajectory rather than a single diagnostic or therapeutic event [7,9]. Recent international consensus criteria have strengthened the conceptual framework for pediatric sepsis and septic shock by centering diagnosis on infection-associated organ dysfunction, while the updated 2026 pediatric Surviving Sepsis Campaign guidance emphasizes that time-sensitive recognition, serial reassessment, and individualized management remain central to care [7,9,10].

From a practical perspective, outcomes are shaped by a sequence of interdependent steps: early recognition in the emergency department, prompt antimicrobial therapy, careful fluid and vasoactive management, vigilant ward monitoring, timely escalation to PICU, and phenotype-informed intensive care support [6,9,34,106]. Delays or limitations at any point in this pathway may contribute to greater organ dysfunction, more intensive resource use, and worse recovery [42,77].

Survival alone does not capture the full burden of pediatric septic shock; organ dysfunction burden, duration of respiratory and vasoactive support, length of PICU and hospital stay, and longer-term functional recovery are also clinically meaningful outcomes [18,70,71,80]. Accordingly, optimizing pediatric septic shock care requires not only better therapies, but also better integration of care across settings and transitions [75,78,79].

Diagnostic and prognostic tools, including lactate trends, organ dysfunction scores, bedside hemodynamic assessment, and emerging prediction models, may increasingly support earlier identification of children at highest risk [19,72,84,107]. However, no single tool can replace expert clinical judgment, repeated bedside evaluation, and careful adaptation of therapy to the child’s evolving physiology [9,24,98].

In summary, pediatric septic shock should be approached through a continuum-of-care model that connects recognition, treatment, escalation, intensive care management, and recovery [7,9,78]. Such an approach may better reflect real-world clinical practice, identify opportunities to reduce preventable deterioration, and ultimately improve both short-term and longer-term outcomes in children with septic shock [78,82,83].

## Figures and Tables

**Table 1:** Summary of contemporary conceptual and bedside principles relevant to pediatric septic shock across the hospital care pathway.

*Concept*	Current Definition/Clinical Meaning	Bedside Implications
*Pediatric sepsis*	Infection-associated organ dysfunction in a child with suspected infection, operationalized by a Phoenix Sepsis Score ≥2	Supports structured recognition of clinically significant sepsis; should complement, not replace, bedside judgment
*Pediatric septic shock*	Pediatric sepsis with cardiovascular dysfunction, including severe age-adjusted hypotension, markedly elevated lactate, or vasoactive requirement	Identifies a high-risk subgroup requiring urgent hemodynamic reassessment, early treatment, and likely PICU-level care
*Organ dysfunction-based framework*	Contemporary definitions emphasize organ dysfunction rather than the older SIRS-centered model	Better reflects severity and prognostic relevance, but may not capture very early evolving shock before full criteria are met
*Dynamic trajectory*	Pediatric septic shock often evolves progressively rather than presenting as an immediately obvious syndrome	Repeated reassessment is essential across ED, ward, and PICU settings
*Hypotension as a late sign*	Children may preserve blood pressure until late because of compensatory vasoconstriction and tachycardia	Absence of hypotension should not reassure clinicians when perfusion is abnormal
*Early perfusion abnormalities*	Abnormal capillary refill, weak pulses, cool extremities, tachycardia, altered mental status, oliguria, or rising lactate may indicate evolving shock	Early treatment decisions should be based on the overall physiologic picture, not on blood pressure alone
*Hemodynamic heterogeneity*	Children may present with vasodilatory, myocardial, mixed, or evolving shock phenotypes	Fixed one-size-fits-all treatment is inappropriate; management should be individualized and repeatedly adapted
*Hospital-trajectory model*	Septic shock may begin in the community or emerge later during hospitalization, including on pediatric, surgical, or oncology wards	Recognition and escalation must be hospital-wide responsibilities, not confined to the ED or PICU

**Table 2:** Practical early management bundle across the three main hospital settings in which pediatric septic shock may be recognized or managed.

*Domain*	ED/First Recognition	Inpatient Ward/Ongoing Deterioration	PICU/Early Intensive Management
*Clinical* *suspicion*	Consider sepsis in children with suspected or confirmed infection plus abnormal perfusion, altered mental status, respiratory distress, or deteriorating vital signs	Reconsider sepsis in children with new or worsening instability, including postoperative, oncology, immunocompromised, or hospital-acquired infection contexts	Confirm the clinical trajectory and reassess shock phenotype, organ dysfunction burden, and response to treatment
*Monitoring*	Perform serial bedside reassessment of perfusion, mental status, heart rate, blood pressure, urine output, respiratory effort, and oxygen requirement	Maintain close surveillance for persistent or progressive organ dysfunction; use repeated reassessment rather than passive observation, supported where appropriate by PEWS or local deterioration systems	Continue frequent reassessment using bedside signs, lactate trends, and advanced monitoring where available, including cardiac/lung point-of-care ultrasound when supported by local expertise
*Antimicrobial therapy*	Start empiric broad-spectrum therapy promptly after recognition; obtain cultures when feasible without clinically important delay	Reassess, escalate, or tailor therapy according to the suspected source, hospital-acquired infection risk, comorbidity, and emerging microbiology	Narrow, redirect, or escalate therapy according to microbiologic results, source control, and clinical response
*Fluid therapy*	Administer cautious, reassessment-guided crystalloid boluses, preferably balanced or buffered crystalloids when available; reassess after each bolus and monitor closely for fluid responsiveness and early signs of overload	Avoid automatic repeated boluses; reassess hemodynamics, respiratory status, hepatomegaly, urine output, and fluid accumulation	Transition from initial resuscitation to individualized fluid stewardship and de-resuscitation when appropriate
*Vasoactive support*	Consider early vasoactive therapy when perfusion remains abnormal despite initial fluid resuscitation	Maintain a low threshold for escalation when persistent tachycardia, abnormal perfusion, oliguria, rising lactate, or respiratory deterioration persists	Titrate vasoactive and/or inotropic support according to the dominant hemodynamic phenotype
*Source control*	Identify the likely infectious source early; consider cultures, imaging, line evaluation, and the need for drainage or surgical review	Reassess for postoperative source, device-related infection, abscess, abdominal source, or other hospital-acquired infection	Integrate antimicrobial tailoring with definitive source control and organ support
*Escalation* *decisions*	Determine whether ED observation, ward admission, or PICU referral is the safest option based on response to initial treatment and overall trajectory	Identify persistent abnormal perfusion, rising lactate, vasoactive requirement or likely need for vasoactive initiation, worsening respiratory distress, oliguria, altered mental status, recurrent fluid requirement, or concern that ward-level monitoring is insufficient; escalate promptly to PICU when closer monitoring or organ support is needed	Provide advanced hemodynamic management and organ support once escalation occurs

**Table 3:** Core PICU management domains in pediatric septic shock, with practical targets and the expected physiologic or clinical impact.

*Management Domain*	Main Objective	Practical Targets/Considerations	Expected Clinical Effect
*Hemodynamic reassessment*	Define the dominant shock phenotype and track response to therapy	Repeated evaluation of perfusion, blood pressure, urine output, lactate trends, bedside echocardiography, cardiac/lung point-of-care ultrasound, and advanced hemodynamic variables when available	More individualized and timely adjustment of therapy
*Vasoactive/inotropic therapy*	Restore adequate tissue perfusion	Match treatment to vasoplegia, myocardial dysfunction, or mixed shock states; titrate to perfusion endpoints rather than blood pressure alone	Improved perfusion, organ blood flow, and stabilization of shock
*Respiratory support*	Reduce oxygen demand and support gas exchange	Use supplemental oxygen, noninvasive support, or invasive ventilation according to the degree of respiratory and hemodynamic compromise	Reduced work of breathing and improved oxygen delivery-demand balance
*Mechanical ventilation strategy*	Provide respiratory support without worsening hemodynamics	Apply a lung-protective approach while monitoring the effects of positive pressure ventilation on preload, afterload, and cardiac output	Improved gas exchange with lower risk of secondary hemodynamic deterioration
*Fluid stewardship*	Limit harmful cumulative fluid burden after initial resuscitation	Reassess maintenance fluids, medication diluents, nutrition-related fluids, capillary leak, and overall fluid balance	Lower risk of edema, respiratory worsening, acute kidney injury, and prolonged organ support
*Metabolism/nutrition support*	Limit catabolism and progressively support energy and protein requirements during critical illness	Initiate enteral nutrition within the first 24–48 h when hemodynamically tolerated; monitor feeding tolerance, gastrointestinal dysfunction, protein adequacy, and non-nutritional calorie burden	Reduced cumulative nutritional deficit, support of metabolic recovery, and attenuation of ongoing catabolic stress
*Renal support*	Manage acute kidney injury, fluid overload, or severe metabolic derangement	Consider diuretics, fluid restriction, or renal replacement therapy when indicated	Better fluid control and support of organ recovery
*Rescue therapies*	Support refractory shock or progressive multiorgan dysfunction	Consider ECMO, transfusion support, and correction of coagulation or metabolic abnormalities according to severity and local expertise	Potential stabilization in selected severe cases

**Table 4:** Key outcomes that should be considered across the entire hospital trajectory of pediatric septic shock, beyond mortality alone.

*Outcome Domain*	What It Reflects	Why It Matters Clinically
*Mortality*	Ultimate short-term survival endpoint	Remains important but does not capture the full burden of disease
*Organ dysfunction burden*	Severity and duration of cardiovascular, respiratory, renal, neurologic, or coagulation dysfunction	Reflects both illness severity and effectiveness of timely recognition and treatment
*Duration of vasoactive support*	Persistence of hemodynamic instability	Indicates severity of shock and resource intensity
*Duration of respiratory support*	Extent of respiratory failure and overall critical illness burden	Linked to lung injury, fluid burden, and prolonged PICU stay
*PICU length of stay*	Intensity and duration of critical care needs	Useful marker of morbidity and resource use
*Hospital length of stay*	Global burden across the full admission	Reflects cumulative effects of recognition, treatment, escalation, and recovery
*Unplanned PICU transfer/delayed escalation*	System performance and recognition timeliness outside the PICU	Highlights the importance of ward surveillance and transitions of care
*Functional recovery*	Quality of recovery after survival	Includes neurodevelopment, physical function, cognition, and emotional well-being
*Post-sepsis morbidity/survivorship*	Longer-term sequelae after discharge	Emphasizes that survival alone is not an adequate endpoint in pediatric septic shock

## Data Availability

Not applicable.

## Abbreviations

The following abbreviations are used in this manuscript:

AKI	Acute kidney injury
AUROC	Area under the receiver operating characteristic curve
CRP	C-reactive protein
CRRT	Continuous renal replacement therapy
ECMO	Extracorporeal membrane oxygenation
ED	Emergency department
IL-6	Interleukin-6
PCT	Procalcitonin
PEWS	Pediatric early warning systems
PICU	Pediatric intensive care unit
PICS-p	Post-intensive care syndrome in pediatrics
ScvO_2_	Central venous oxygen saturation
SIRS	Systemic inflammatory response syndrome
sTREM-1	Soluble triggering receptor expressed on myeloid cells-1
SSC	Surviving Sepsis Campaign
suPAR	Soluble urokinase plasminogen activator receptor
TAMOF	Thrombocytopenia-associated multiple organ failure

## References

[1] Zimmerman J.J., Banks R., Berg R.A., Zuppa A., Newth C.J., Wessel D., Pollack M.M., Meert K.L., Hall M.W., Quasney M. (2020). Trajectory of Mortality and Health-Related Quality of Life Morbidity Following Community-Acquired Pediatric Septic Shock. Crit. Care Med..

[2] Watson R.S., Carrol E.D., Carter M.J., Kissoon N., Ranjit S., Schlapbach L.J. (2024). The Burden and Contemporary Epidemiology of Sepsis in Children. Lancet Child. Adolesc. Health.

[3] Scott H.F., Sevick C.J., Colborn K.L., Deakyne Davies S.J., Greer C.H., Dafoe A., Dorsey Holliman B., Bajaj L., Schmidt S.K., Kempe A. (2025). Clinical Decision Support for Septic Shock in the Emergency Department: A Cluster Randomized Trial. Pediatrics.

[4] Ma L., Cao Z., Peng H., Gu X., Lu X., Zhang X., Zhang L. (2026). The Prognostic Value of Capillary Refill Time in Patients with Sepsis: A Prospective Cohort Study. Nurs. Crit. Care.

[5] Du G., Huang H., Zhang R., Liu C., Fu Y. (2026). Role of Cytokine Levels in Pathogen Classification and Prognosis of Pediatric Septic Shock. Front. Immunol..

[6] Oruganti S., Evans J., Cromarty T., Javaid A., Roland D. (2022). Identification of Sepsis in Paediatric Emergency Departments: A Scoping Review. Acta Paediatr..

[7] Schlapbach L.J., Watson R.S., Sorce L.R., Argent A.C., Menon K., Hall M.W., Akech S., Albers D.J., Alpern E.R., Balamuth F. (2024). International Consensus Criteria for Pediatric Sepsis and Septic Shock. JAMA.

[8] Li W., Ge H., Zhang J., Li N., Lu X., Chen J., Qu D., Liu S., Liu C. (2025). Comparison of 2005 and 2024 Diagnostic Criteria for Early Identification of Pediatric Sepsis and Septic Shock in PICU Patients: A Prospective Cohort Study. Front. Pediatr..

[9] Weiss S.L., Peters M.J., Alhazzani W., Agus M.S.D., Flori H.R., Inwald D.P., Nadel S., Schlapbach L.J., Tasker R.C., Argent A.C. (2020). Surviving Sepsis Campaign International Guidelines for the Management of Septic Shock and Sepsis-Associated Organ Dysfunction in Children. Pediatr. Crit. Care Med..

[10] Weiss S.L., Peters M.J., Oczkowski S.J.W., Belley-Cote E., Buysse C., Choong K.L.M., Deep A., Inwald D.P., Flori H.R., Kneyber M.C.J. (2026). Surviving Sepsis Campaign International Guidelines for the Management of Sepsis and Septic Shock in Children 2026. Pediatr. Crit. Care Med..

[11] Beltrán J.C., Fernández-Sarmiento J., Orjuela-Cañón A.D., Sanchez-Pinto L.N., Fernández-Sarta J.P., Rotta I.L., Fernández-Sarta D., Coutin A., Godoy J.E., Vink H. (2026). Microvascular Phenotypes in Pediatric Sepsis Identified by Machine Learning: Prognostic Implications for Organ Dysfunction and Mortality. Crit. Care.

[12] Miliaraki M., Briassoulis G., Dardamani E., Briassoulis P., Ilia S. (2026). Septic Cardiomyopathy: Age-Dependent Physiology and Hemodynamic Aspects-A Narrative Review. Children.

[13] Briassoulis G., Varda S., Marinopoulou E., Kondili E., Ilia S. (2026). Fluid Overload, Renal Angina Index, and PICU Outcomes: Single-Center Retrospective Cohort, 2020-2024. Pediatr. Crit. Care Med..

[14] Du C., Tan S.C., Bu H.-F., Subramanian S., Geng H., Wang X., Xie H., Wu X., Zhou T., Liu R. (2024). Predicting Patients with Septic Shock and Sepsis through Analyzing Whole-Blood Expression of NK Cell-Related Hub Genes Using an Advanced Machine Learning Framework. Front. Immunol..

[15] Blankenship L., Demmel K., Otis T. (2024). Sepsis Education and Successful Implementation of a Sepsis Recognition and Management Workflow in an Inpatient Pediatric Hematologic Oncologic Unit. J. Pediatr. Hematol. Oncol. Nurs..

[16] Weiss S.L., Fitzgerald J.C., Pappachan J., Wheeler D., Jaramillo-Bustamante J.C., Salloo A., Singhi S.C., Erickson S., Roy J.A., Bush J.L. (2015). Global Epidemiology of Pediatric Severe Sepsis: The Sepsis Prevalence, Outcomes, and Therapies Study. Am. J. Respir. Crit. Care Med..

[17] Phumeetham S., Limprayoon K., Law S., Preeprem N., Kriengsoontornkij W. (2025). A Quality Improvement Intervention for the Initial Care of Pediatric Septic Shock in a Resource-Limited Setting. J. Pediatr. Clin. Pract..

[18] Sobotka S.A., Lynch E.J., Pinto N.P. (2025). Three-Year Follow-Up of PICU Survivors: Time Course of Neurodevelopmental Sequelae in a Single-Center Cohort, Recruited 2017-2018. Pediatr. Crit. Care Med..

[19] de Souza D.C., Jabornisky R., Kissoon N. (2024). Utility of Lactate Levels in the Diagnosis and Prognosis of Septic Shock. Pediatr. Emerg. Care.

[20] Goldstein B., Giroir B., Randolph A. (2005). International Consensus Conference on Pediatric Sepsis International Pediatric Sepsis Consensus Conference: Definitions for Sepsis and Organ Dysfunction in Pediatrics. Pediatr. Crit. Care Med..

[21] Shamavu G.K., Mohamoud F. (2025). Rethinking Pediatric Sepsis and Septic Shock: Beyond International Consensus Criteria. Pediatr. Health Med. Ther..

[22] Toltzis P., Remy K.E. (2024). New Criteria for Pediatric Sepsis: A Phoenix Rising. J. Pediatr. Pharmacol. Ther..

[23] Ranjit S., Natraj R., Kissoon N., Thiagarajan R.R., Ramakrishnan B., Monge García M.I. (2021). Variability in the Hemodynamic Response to Fluid Bolus in Pediatric Septic Shock. Pediatr. Crit. Care Med..

[24] Ranjit S., Kissoon N., Argent A., Inwald D., Ventura A.M.C., Jaborinsky R., Sankar J., de Souza D.C., Natraj R., De Oliveira C.F. (2023). Haemodynamic Support for Paediatric Septic Shock: A Global Perspective. Lancet Child. Adolesc. Health.

[25] Zampieri F.G., Damiani L.P., Bakker J., Ospina-Tascón G.A., Castro R., Cavalcanti A.B., Hernandez G. (2020). Effects of a Resuscitation Strategy Targeting Peripheral Perfusion Status versus Serum Lactate Levels among Patients with Septic Shock. A Bayesian Reanalysis of the ANDROMEDA-SHOCK Trial. Am. J. Respir. Crit. Care Med..

[26] Fernández-Sarmiento J., Lamprea S., Barrera S., Acevedo L., Duque C., Trujillo M., Aguirre V., Jimenez C. (2024). The Association between Prolonged Capillary Refill Time and Microcirculation Changes in Children with Sepsis. BMC Pediatr..

[27] Georgette N., Michelson K., Monuteaux M., Eisenberg M.A. (2025). Comparing Screening Tools for Predicting Phoenix Criteria Sepsis and Septic Shock Among Children. Pediatrics.

[28] Gawronski O., Briassoulis G., El Ghannudi Z., Ilia S., Sánchez-Martín M., Chiusolo F., Jensen C.S., Manning J.C., Valla F.V., Pavelescu C. (2024). European Survey on Paediatric Early Warning Systems, and Other Processes Used to Aid the Recognition and Response to Children’s Deterioration on Hospital Wards. Nurs. Crit. Care.

[29] Gómez-Ramos J.J., Marín-Medina A., Prieto-Miranda S.E., Dávalos-Rodríguez I.P., Alatorre-Jiménez M.A., Esteban-Zubero E. (2018). Determination of Plasma Lactate in the Emergency Department for the Early Detection of Tissue Hypoperfusion in Septic Patients. Am. J. Emerg. Med..

[30] Souganidis E.S., Patel B., Sampayo E.M. (2022). Physician-Specific Utilization of an Electronic Best Practice Alert for Pediatric Sepsis in the Emergency Department. Pediatr. Emerg. Care.

[31] Martinez E.M., Sepanski R.J., Jennings A.D., Schmidt J.M., Cholis T.J., Dominy M.E., Devlin S.B., Eilers L.F., Zaritsky A.L., Godambe S.A. (2023). Optimizing Recognition and Management of Patients at Risk for Infection-Related Decompensation Through Team-Based Decision Making. J. Healthc. Qual..

[32] Park H., Lee J., Oh D.K., Park M.H., Lim C.-M., Lee S.-M., Lee H.Y. (2023). Korean Sepsis Alliance (KSA) Investigators Serial Evaluation of the Serum Lactate Level with the SOFA Score to Predict Mortality in Patients with Sepsis. Sci. Rep..

[33] Park C., Ku N.S., Park D.W., Park J.H., Ha T.S., Kim D.W., Park S.Y., Chang Y., Jo K.W., Baek M.S. (2024). Early Management of Adult Sepsis and Septic Shock: Korean Clinical Practice Guidelines. Acute Crit. Care.

[34] Weiss S.L., Fitzgerald J.C. (2024). Pediatric Sepsis Diagnosis, Management, and Sub-Phenotypes. Pediatrics.

[35] Inwald D., Canter R.R., Woolfall K., O’Hara C.B., Mouncey P.R., Zenasni Z., Hudson N., Saunders S., Carter A., Jones N. (2018). Restricted Fluid Bolus versus Current Practice in Children with Septic Shock: The Fish Feasibility Study and Pilot RCT. Health Technol. Assess..

[36] Briassoulis G., Antonopoulou T., Velegraki J., Ilia S., Kondili E. (2025). Fluid Creep as an Independent Predictor of Fluid Overload and Mortality in Critically Ill Patients: A Cohort Study. Life.

[37] Harley A., George S., Phillips N., King M., Long D., Keijzers G., Lister P., Raman S., Bellomo R., Gibbons K. (2024). Resuscitation with Early Adrenaline Infusion for Children with Septic Shock: A Randomized Pilot Trial. Pediatr. Crit. Care Med..

[38] Shi R., Braïk R., Monnet X., Gu W.-J., Ospina-Tascon G., Permpikul C., Djebbour M., Soumare A., Agaleridis V., Lai C. (2025). Early Norepinephrine for Patients with Septic Shock: An Updated Systematic Review and Meta-Analysis with Trial Sequential Analysis. Crit. Care.

[39] Tabah A., De Waele J., Ssi Yan Kai N., Aslan A.T., Buetti N., Timsit J.-F., Ballard E., Eriksson L., Laupland K.B., Lipman J. (2025). Source Control in Bloodstream Infections in Patients with Sepsis, Septic Shock, or Requiring ICU Admission: A Scoping Review with Recommendations for Standardizing Research. Intensive Care Med..

[40] McElroy T., Swartz E.N., Hassani K., Waibel S., Tuff Y., Marshall C., Chan R., Wensley D., O’Donnell M. (2019). Implementation Study of a 5-Component Pediatric Early Warning System (PEWS) in an Emergency Department in British Columbia, Canada, to Inform Provincial Scale Up. BMC Emerg. Med..

[41] Alsabri M., Mourid M.R., Saleh A.R., Oyedele T.J., Ata I.M., Darawish S.M., Patel A., Rouh F.A., Carr L.A. (2026). Vital Signs as Biomarkers of Early Clinical Deterioration in Pediatric Emergency Departments: Physiology, Interpretation, and Innovations: A Narrative Review. Int. J. Emerg. Med..

[42] Scott H.F., Lindberg D.M., Brackman S., McGonagle E., Leonard J.E., Adelgais K., Bajaj L., Dillon M., Kempe A. (2024). Pediatric Sepsis in General Emergency Departments: Association between Pediatric Sepsis Case Volume, Care Quality, and Outcome. Ann. Emerg. Med..

[43] Allen D., Lloyd A., Edwards D., Hood K., Huang C., Hughes J., Jacob N., Lacy D., Moriarty Y., Oliver A. (2022). Development, Implementation and Evaluation of an Evidence-Based Paediatric Early Warning System Improvement Programme: The PUMA Mixed Methods Study. BMC Health Serv. Res..

[44] Alcamo A.M., Lindell R.B., Sheetz S.A., Ham S.D., Strayer A., Weiss S.L., Nishisaki A., Pinto N.P., Topjian A.A., Fitzgerald J.C. (2025). New Sepsis-Associated Morbidity and Mortality in Pediatric Oncology Patients. Front. Oncol..

[45] Hakim H., Richardson T., Riggs R., Auletta J.J., DiGerolamo K., Hron J.D., Kohorst M., Laurie K., Maixner M., Mulcahy Levy J.M. (2025). Sepsis Mortality in Hospitalized Children with Cancer Is Associated with Lack of a Screening Tool. Hosp. Pediatr..

[46] Al-Harbi S. (2024). Impact of Rapid Response Teams on Pediatric Care: An Interrupted Time Series Analysis of Unplanned PICU Admissions and Cardiac Arrests. Healthcare.

[47] Walker S.B., Lockwood J.M., Scott H.F., Sanchez-Pinto L.N., Bennett T.D. (2025). We Have New Sepsis Criteria for Children…Now What?. Hosp. Pediatr..

[48] Agulnik A., Muniz-Talavera H., Pham L.T.D., Chen Y., Carrillo A.K., Cárdenas-Aguirre A., Gonzalez Ruiz A., Garza M., Conde Morelos Zaragoza T.M., Soberanis Vasquez D.J. (2023). Effect of Paediatric Early Warning Systems (PEWS) Implementation on Clinical Deterioration Event Mortality among Children with Cancer in Resource-Limited Hospitals in Latin America: A Prospective, Multicentre Cohort Study. Lancet Oncol..

[49] Lin K., Weng X., Du B., Tian T., Quan X. (2025). Analysis of the Accuracy of Disease Prediction in Pediatric Ward Patients Based on the Modified Early Warning Score for Children: A Randomized Controlled Trial. Heliyon.

[50] Bracken A., Lane S., Siner S., Jones D., Lambert C., Mehta F., Eyton-Chong C.-K., Davis P., Fitzsimons J., Lim E. (2025). Assessing the Performance of Paediatric Early Warning Scores to Predict Critical Deterioration Events in Hospitalised Children (the DETECT Study): A Retrospective Matched Case-Control Study. BMC Pediatr..

[51] Caraballo C., Jaimes F. (2019). Organ Dysfunction in Sepsis: An Ominous Trajectory from Infection to Death. Yale J. Biol. Med..

[52] Lee J., Ciuchta J.L., Weingarten-Arams J., Philips K. (2024). Pediatric Early Warning Scores Before Rapid Response Poorly Predict Intensive Care Unit Transfers. Hosp. Pediatr..

[53] Emeriaud G., López-Fernández Y.M., Iyer N.P., Bembea M.M., Agulnik A., Barbaro R.P., Baudin F., Bhalla A., Brunow de Carvalho W., Carroll C.L. (2023). Executive Summary of the Second International Guidelines for the Diagnosis and Management of Pediatric Acute Respiratory Distress Syndrome (PALICC-2). Pediatr. Crit. Care Med..

[54] Hsu B.S., Hill V., Frankel L.R., Yeh T.S., Simone S., Arca M.J., Coss-Bu J.A., Fallat M.E., Foland J., Gadepalli S. (2019). Executive Summary: Criteria for Critical Care of Infants and Children: PICU Admission, Discharge, and Triage Practice Statement and Levels of Care Guidance. Pediatrics.

[55] Demirer Aydemir F., Kurtkulagi O., Ergun B., Bayrak V., Oner O., Comert B., Gokmen A.N., Hanci V. (2025). ICU Admission Delays: Impact on Length of Stay and Long-Term Outcomes. Biomol. Biomed..

[56] Chanci D., Grunwell J.R., Rafiei A., Brown S.R., Ripple M.J., Bishop N.R., Rajapreyar P., Lima L.M., Kamaleswaran R. (2026). Machine Learning Model for Daily Prediction of Pediatric Sepsis Using Phoenix Criteria. Pediatr. Res..

[57] Davis A.L., Carcillo J.A., Aneja R.K., Deymann A.J., Lin J.C., Nguyen T.C., Okhuysen-Cawley R.S., Relvas M.S., Rozenfeld R.A., Skippen P.W. (2017). American College of Critical Care Medicine Clinical Practice Parameters for Hemodynamic Support of Pediatric and Neonatal Septic Shock. Crit. Care Med..

[58] Lee E.-P., Wu H.-P., Chan O.-W., Lin J.-J., Hsia S.-H. (2022). Hemodynamic Monitoring and Management of Pediatric Septic Shock. Biomed. J..

[59] Williams F.Z., Sachdeva R., Travers C.D., Walson K.H., Hebbar K.B. (2019). Characterization of Myocardial Dysfunction in Fluid- and Catecholamine-Refractory Pediatric Septic Shock and Its Clinical Significance. J. Intensive Care Med..

[60] Kong X., Zhu Y., Zhu X. (2021). Association between Early Fluid Overload and Mortality in Critically-Ill Mechanically Ventilated Children: A Single-Center Retrospective Cohort Study. BMC Pediatr..

[61] Eisenberg M.A., Georgette N., Baker A.H., Priebe G.P., Monuteaux M.C. (2025). Epinephrine vs Norepinephrine as Initial Treatment in Children with Septic Shock. JAMA Netw. Open.

[62] Weiss S.L. (2025). Vasoactive Selection for Pediatric Septic Shock-Where to Begin?. JAMA Netw. Open.

[63] Asiri N., Bahatheq L.K., Shaheen N., Kazzaz Y.M. (2026). Burden and Outcomes of Pediatric Acute Respiratory Distress Syndrome among Children with Sepsis: A Cohort Study. Front. Pediatr..

[64] Khemani R.G., Smith L., Lopez-Fernandez Y.M., Kwok J., Morzov R., Klein M.J., Yehya N., Willson D., Kneyber M.C.J., Lillie J. (2019). Paediatric Acute Respiratory Distress Syndrome Incidence and Epidemiology (PARDIE): An International, Observational Study. Lancet Respir. Med..

[65] Wong J.J.M., Dang H., Gan C.S., Phan P.H., Kurosawa H., Aoki K., Lee S.W., Ong J.S.M., Fan L.J., Tai C.W. (2024). Lung-Protective Ventilation for Pediatric Acute Respiratory Distress Syndrome: A Nonrandomized Controlled Trial. Crit. Care Med..

[66] Díaz F., Nuñez M.J., Pino P., Erranz B., Cruces P. (2018). Implementation of Preemptive Fluid Strategy as a Bundle to Prevent Fluid Overload in Children with Acute Respiratory Distress Syndrome and Sepsis. BMC Pediatr..

[67] Srouji L.S., Moore-Clingenpeel M., Hensley J., Steele L., Greathouse K., Anglim L., Hanson-Huber L., Nateri J., Nicol K., Hall M.W. (2020). Shock Severity Modifies Associations Between RBC Transfusion in the First 48 Hours of Sepsis Onset and the Duration of Organ Dysfunction in Critically Ill Septic Children. Pediatr. Crit. Care Med..

[68] Yang J.O., Zinter M.S., Pellegrini M., Wong M.Y., Gala K., Markovic D., Nadel B., Peng K., Do N., Mangul S. (2023). Whole Blood Transcriptomics Identifies Subclasses of Pediatric Septic Shock. Crit. Care.

[69] Dunwoodie L., Huang M., Moore A.R., Stanski N.L., Standage S.W., Kaplan J.M., Zingarelli B., Harmon K., Fitzgerald J.C., Weiss S.L. (2025). Neutrophil Dysregulation Differentiates Pediatric Septic Shock Biomarker-Based Mortality-Risk Strata: Insights from Weighted Gene Co-Expression Network and Transcriptomic Analyses. Front. Immunol..

[70] Workman J.K., Reeder R.W., Banks R.K., Zimmerman J.J., Meert K.L., Keenan H.T. (2023). Change in Functional Status During Hospital Admission and Long-Term Health-Related Quality of Life Among Pediatric Septic Shock Survivors. Pediatr. Crit. Care Med..

[71] Maddux A.B., Zimmerman J.J., Banks R.K., Reeder R.W., Meert K.L., Czaja A.S., Berg R.A., Sapru A., Carcillo J.A., Newth C.J.L. (2022). Health Resource Use in Survivors of Pediatric Septic Shock in the United States. Pediatr. Crit. Care Med..

[72] Sanchez-Pinto L.N., Daniels L.A., Atreya M., Faustino E.V.S., Farris R.W.D., Geva A., Khemani R.G., Rogerson C., Shah S.S., Weiss S.L. (2025). Phoenix Sepsis Criteria in Critically Ill Children: Retrospective Validation Using a United States Nine-Center Dataset, 2012-2018. Pediatr. Crit. Care Med..

[73] Menon K., Schlapbach L.J., Akech S., Argent A., Biban P., Carrol E.D., Chiotos K., Jobayer Chisti M., Evans I.V.R., Inwald D.P. (2022). Criteria for Pediatric Sepsis-A Systematic Review and Meta-Analysis by the Pediatric Sepsis Definition Taskforce. Crit. Care Med..

[74] Pollack M.M., Holubkov R., Reeder R., Dean J.M., Meert K.L., Berg R.A., Newth C.J.L., Berger J.T., Harrison R.E., Carcillo J. (2018). PICU Length of Stay: Factors Associated with Bed Utilization and Development of a Benchmarking Model. Pediatr. Crit. Care Med..

[75] Dervan L.A., Hartman M., Fink E.L., Fitzgerald J.C., Hall T.A., Laux K., Morgan L.A., Murphy S., Pinto N.P., Schrock E. (2025). Eight PICU Follow-Up Programs in the United States Established From 2013 to 2022: Report from the Pediatric Outcomes Studies After PICU (POST-PICU) Investigators. Pediatr. Crit. Care Med..

[76] Minogue J., Keogh S., Schlapbach L.J., Long D. (2024). Long-Term Outcomes after Paediatric Sepsis: A Narrative Review. Aust. Crit. Care.

[77] Kamidani R., Okada H. (2025). Centralization and Transport of Critically Ill Pediatric Patients. Front. Pediatr..

[78] Vidrine R., Atreya M.R., Stalets E.L. (2018). Continuum of Care in Pediatric Sepsis: A Prototypical Acute Care Delivery Model. Transl. Pediatr..

[79] Larsen G.Y., Brilli R., Macias C.G., Niedner M., Auletta J.J., Balamuth F., Campbell D., Depinet H., Frizzola M., Hueschen L. (2021). Development of a Quality Improvement Learning Collaborative to Improve Pediatric Sepsis Outcomes. Pediatrics.

[80] Ravikumar N., Sankar J., Das R.R. (2022). Functional Outcomes in Survivors of Pediatric Sepsis: A Scoping Review and Discussion of Implications for Low- and Middle-Income Countries. Front. Pediatr..

[81] Koutroulis I., Velez T., Wang T., Yohannes S., Galarraga J.E., Morales J.A., Freishtat R.J., Chamberlain J.M. (2022). Pediatric Sepsis Phenotypes for Enhanced Therapeutics: An Application of Clustering to Electronic Health Records. J. Am. Coll. Emerg. Physicians Open.

[82] Atreya M.R., Huang M., Moore A.R., Zheng H., Hasin-Brumshtein Y., Fitzgerald J.C., Weiss S.L., Cvijanovich N.Z., Bigham M.T., Jain P.N. (2023). Derivation, Validation, and Transcriptomic Assessment of Pediatric Septic Shock Phenotypes Identified Through Latent Profile Analyses: Results from a Prospective Multi-Center Observational Cohort. Res. Sq..

[83] Qin Y., Kernan K.F., Fan Z., Park H.-J., Kim S., Canna S.W., Kellum J.A., Berg R.A., Wessel D., Pollack M.M. (2022). Machine Learning Derivation of Four Computable 24-h Pediatric Sepsis Phenotypes to Facilitate Enrollment in Early Personalized Anti-Inflammatory Clinical Trials. Crit. Care.

[84] Tennant R., Graham J., Kern J., Mercer K., Ansermino J.M., Burns C.M. (2024). A Scoping Review on Pediatric Sepsis Prediction Technologies in Healthcare. NPJ Digit. Med..

[85] Luka S., Golea A., Tat R.M., Lupan Mureșan E.M., Voicescu G.T., Vesa Ș.C., Ionescu D. (2024). Biomarkers as Predictors of Mortality in Sepsis and Septic Shock for Patients Admitted to Emergency Department: Who Is the Winner? A Prospective Study. J. Clin. Med..

[86] Smok B., Domagalski K., Pawłowska M. (2020). Diagnostic and Prognostic Value of IL-6 and sTREM-1 in SIRS and Sepsis in Children. Mediat. Inflamm..

[87] Chen M., Zhu Y. (2020). Utility of sTREM-1 and Presepsin (sCD14-ST) as Diagnostic and Prognostic Markers of Sepsis. Clin. Lab..

[88] Miliaraki M., Briassoulis P., Ilia S., Polonifi A., Mantzourani M., Briassouli E., Vardas K., Nanas S., Pistiki A., Theodorakopoulou M. (2021). Survivin and Caspases Serum Protein Levels and Survivin Variants mRNA Expression in Sepsis. Sci. Rep..

[89] Papadopoulos P., Pistiki A., Theodorakopoulou M., Christodoulopoulou T., Damoraki G., Goukos D., Briassouli E., Dimopoulou I., Armaganidis A., Nanas S. (2017). Immunoparalysis: Clinical and Immunological Associations in SIRS and Severe Sepsis Patients. Cytokine.

[90] Fitrolaki M.-D., Dimitriou H., Venihaki M., Katrinaki M., Ilia S., Briassoulis G. (2016). Increased Extracellular Heat Shock Protein 90α in Severe Sepsis and SIRS Associated with Multiple Organ Failure and Related to Acute Inflammatory-Metabolic Stress Response in Children. Medicine.

[91] Spanaki A.M., Tavladaki T., Dimitriou H., Kozlov A.V., Duvigneau J.C., Meleti E., Weidinger A., Papakonstantinou E., Briassoulis G. (2018). Longitudinal Profiles of Metabolism and Bioenergetics Associated with Innate Immune Hormonal Inflammatory Responses and Amino-Acid Kinetics in Severe Sepsis and Systemic Inflammatory Response Syndrome in Children. JPEN J. Parenter. Enter. Nutr..

[92] Vardas K., Ilia S., Sertedaki A., Charmandari E., Briassouli E., Goukos D., Apostolou K., Psarra K., Botoula E., Tsagarakis S. (2017). Increased Glucocorticoid Receptor Expression in Sepsis Is Related to Heat Shock Proteins, Cytokines, and Cortisol and Is Associated with Increased Mortality. Intensive Care Med. Exp..

[93] Miliaraki M., Briassoulis P., Ilia S., Michalakakou K., Karakonstantakis T., Polonifi A., Bastaki K., Briassouli E., Vardas K., Pistiki A. (2022). Oxidant/Antioxidant Status Is Impaired in Sepsis and Is Related to Anti-Apoptotic, Inflammatory, and Innate Immunity Alterations. Antioxidants.

[94] Ye Q., Lai X., Liu Y., Zhang Z., Fu Y., Luo J., Liu C., Duan J., Ding H., Liu Y. (2026). Single-Cell Multi-Omic Landscape Reveals Anatomical-Specific Immune Features in Adult and Pediatric Sepsis. Nat. Immunol..

[95] Briassoulis G., Briassoulis P., Miliaraki M., Ilia S., Parlato M., Philippart F., Rouquette A., Moucadel V., Cavaillon J.-M., Misset B. (2019). Biomarker Cruises in Sepsis: Who Is the CAPTAIN? Discussion on “Circulating Biomarkers May Be Unable to Detect Infection at the Early Phase of Sepsis in ICU Patients: The CAPTAIN Prospective Multicenter Cohort Study”. Intensive Care Med..

[96] Song Y., Wang N., Xie X., Tian Y., Wang Y. (2024). Relationship between Lactate Levels and 28-Day Mortality in Pediatric Sepsis: Results from the Pediatric Intensive Care Database. BMC Pediatr..

[97] Tavladaki T., Spanaki A.M., Dimitriou H., Kondili E., Choulaki C., Georgopoulos D., Briassoulis G. (2017). Similar Metabolic, Innate Immunity, and Adipokine Profiles in Adult and Pediatric Sepsis Versus Systemic Inflammatory Response Syndrome-A Pilot Study. Pediatr. Crit. Care Med..

[98] Esposito S., Mucci B., Alfieri E., Tinella A., Principi N. (2025). Advances and Challenges in Pediatric Sepsis Diagnosis: Integrating Early Warning Scores and Biomarkers for Improved Prognosis. Biomolecules.

[99] Sanchez-Pinto L.N., Bennett T.D., DeWitt P.E., Russell S., Rebull M.N., Martin B., Akech S., Albers D.J., Alpern E.R., Balamuth F. (2024). Development and Validation of the Phoenix Criteria for Pediatric Sepsis and Septic Shock. JAMA.

[100] Long E., Borland M.L., George S., Jani S., Tan E., Phillips N., Kochar A., Craig S., Lithgow A., Rao A. (2025). External Validation of the Phoenix Sepsis Score in Children with Suspected Community-Acquired Sepsis. JAMA Netw. Open.

[101] Arnoldi S., Glau C.L., Walker S.B., Himebauch A.S., Parikh D.S., Udeh S.C., Weiss S.L., Fitzgerald J.C., Nishisaki A., Conlon T.W. (2021). Integrating Focused Cardiac Ultrasound into Pediatric Septic Shock Assessment. Pediatr. Crit. Care Med..

[102] El-Zayat R.S., Shalaby A.G. (2018). Mitral Annular Plane Systolic Excursion as a Predictor of Mortality in Children with Septic Shock. Pediatr. Crit. Care Med..

[103] Reveco S., Barbagelata S., Cruces P., Diaz F., Yohanessen K., Larraín M., Guerra M., Bataszew A. (2025). Functional Echocardiography Identifies Association between Early Ventricular Dysfunction and Outcome in Pediatric Sepsis. Front. Pediatr..

[104] Sanchez-Pinto L.N., Del Pilar Arias López M., Scott H., Gibbons K., Moor M., Watson R.S., Wiens M.O., Schlapbach L.J., Bennett T.D. (2024). Digital Solutions in Paediatric Sepsis: Current State, Challenges, and Opportunities to Improve Care around the World. Lancet Digit. Health.

[105] Mann K.D., Good N.M., Fatehi F., Khanna S., Campbell V., Conway R., Sullivan C., Staib A., Joyce C., Cook D. (2021). Predicting Patient Deterioration: A Review of Tools in the Digital Hospital Setting. J. Med. Internet Res..

[106] Aldewereld Z., Horvat C., Carcillo J.A., Clermont G. (2024). EMERGENCE OF A TECHNOLOGY-DEPENDENT PHENOTYPE OF PEDIATRIC SEPSIS IN A LARGE CHILDREN’S HOSPITAL. Shock.

[107] Stephen R.J., Carroll M.S., Hoge J., Maciorowski K., Jones R.C., Lucey K., O’Connell M., Schwab C., Rojas J., Sanchez-Pinto L.N. (2023). Sepsis Prediction in Hospitalized Children: Model Development and Validation. Hosp. Pediatr..

